# Non-perforated Stercoral Colitis patients with septic shock have a higher mortality than their perforated counterparts. A case report and review of literature

**DOI:** 10.1016/j.ijscr.2022.107528

**Published:** 2022-08-17

**Authors:** Cesar Reategui, Derek Grubbs

**Affiliations:** Department of Surgery, Missouri Delta Medical Center, Sikeston, MO, USA

**Keywords:** Case report, Stercoral Colitis, Sepsis, Mortality

## Abstract

**Introduction and importance:**

Stercoral colitis is an inflammatory condition caused by fecal impaction; it involves the colonic or rectal wall. It occurs most commonly in nursing home patients, chronic opioid users, and patients with mental impairment.

**Case presentation:**

We present the case of a 36-year-old, obese, African American male with a history of intellectual disability, bipolar disorder, and chronic constipation. Patient presented to the emergency room after an episode of syncope, confusion, 24-hour abdominal pain, nausea, and vomiting. On admission to the ED the patient was found to be in sepsis; within 4 h he developed septic shock. CT scan of the abdomen showed impacted fecal matter in a significantly distended left and sigmoid colon. This was associated with colitis, extensive fat stranding and free fluid, without pneumoperitoneum. The patient was taken to the operating room for exploration where he underwent an extended left colectomy and Hartmann's procedure. Pathology showed acute focal colitis with transmural necrosis. There were no signs of perforation or inflammatory bowel disease. The patient recovered and was discharged home on post-operative day 8. Upon follow up on post-operative day 22, he was doing well.

**Clinical discussion:**

This case illustrates a very rare and challenging scenario. Complications of stercoral colitis include: stercoral ulcer, perforation, ischemic colitis, sepsis and death. Peritonitis, sepsis and bowel necrosis without perforation is extremely rare with very few cases reported in the literature. Colectomy with diversion is the mainstay of therapy.

**Conclusion:**

It is of paramount importance for ED providers and general surgeons to be aware of this condition. It presents a diagnostic challenge and carries an elevated mortality. Elderly patients on chronic opioids and those with mental impairment are at a higher risk.

## Highlights

• Stercoral colitis induced sepsis is a challenging diagnosis.
• Stercoral colitis induced sepsis has a mortality close to 60 %.
• Source control is paramount to avoid fatal outcomes.

## 1 Introduction and importance

Stercoral Colitis (SC) is an unusual condition caused by fecal impaction. It usually affects the sigmoid colon and the rectum. Nursing home patients, chronic opioid users, and patients with mental impairment most commonly develop SC. Complications of SC include: stercoral ulcer, perforation, ischemic colitis, sepsis, septic shock, and death [1]. Perforated SC (PSC) is uncommon. Non-perforated SC (NPSC) presenting with sepsis and colonic transmural necrosis is much less common. Its management depends on the clinical scenario. Patients with signs of peritoneal irritation on exam require surgical intervention likely along with bowel resection. The decision for anastomosis, diversion or damage control depends on the clinical picture [2]. We present the case of a 36-year-old male African American patient with SC who presented to the emergency department (ED) with an acute abdomen progressing rapidly to septic shock. He underwent extended left hemicolectomy and Hartmann's procedure. Pathology showed colonic transmural necrosis without perforation or signs of inflammatory bowel disease (IBD). This case report is in line with the SCARE 2020 criteria [3].

## 2 Case presentation

A 36-year-old obese, African American male presented to the ED with a 24-hour history of diffuse abdominal pain after a syncopal episode. Symptoms included weakness, mental status changes, and worsening constipation. Medical history was significant for moderate intellectual disability, bipolar disorder, hypertension, constipation, and obstructive sleep apnea. Surgical history included a tonsillectomy. Home medications included: diltiazem, hydrochlorothiazide, lisinopril, citalopram, divalproex sodium, bupropion SR, benztropine, clonazepam, and Haldol Decanoate. He had no significant family history.

Initial vital signs (VS) showed: blood pressure (BP) 95/64 mmHg, heart rate (HR) 112 bpm. Upon physical exam, the patient was awake, alert, in no distress, with a distended and diffusely tender abdomen. Initial lab results can be seen on Table 1, showing leukocytosis, lactic acidosis, and elevated creatinine.

**Table 1:** Admission laboratory work up.

Admission laboratory results	
WBC	(H) 14.9 × 10^3^/mcL
Auto neutrophil %	(H) 79.2 %
Hgb	15.2 g/dL
Platelets	296 × 10^3^/mcL
Chloride	(H) 109 mmol/L
CO_2_	(L) 11 mmol/L
Anion gap	(H) 22.8 mmol/L
BUN	(H) 21 mg/dL
Creatinine	(H) 2.0 mg/dL
Calcium	(L) 7.1 mg/dL
Bili total	0.8 mg/dL
ALT	41 kunits/L
Lactic acid	(H) 10.7 mmol/L
Procalcitonin	(H) 63.66 ng/mL

**Fig. 1:**
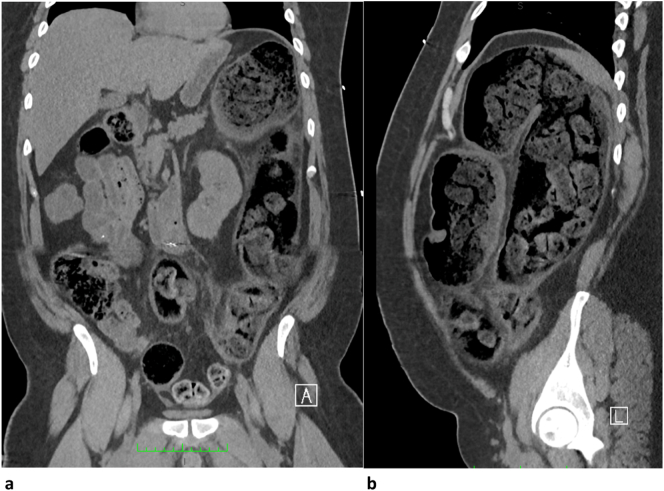
Coronal (a) and sagittal (b) views showing fecal impaction of the splenic flexure and descending colon. Wall thickening can be appreciated in both images.

**Fig. 2:**
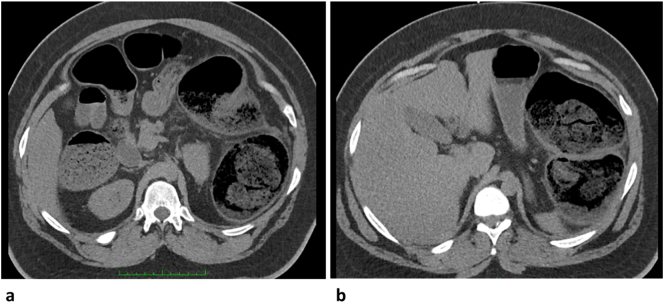
Axial view in panel a showing circumferential wall thickening of the hepatic flexure and proximal transverse colon with marked luminal narrowing. Panel b showing axial view of impacted and dilated splenic flexure.

Abdomen and pelvic CT scans (Fig. 1, Fig. 2) were performed without IV contrast given his acute kidney injury. It reported:

1.- Segmental thickening of the hepatic flexure and proximal transverse colon, would be related to focal colitis with associated stricture; however neoplastic etiology cannot be excluded.
2.- Stercoral colitis involving the splenic flexure and descending colon due to impacted feces.
3.- Pericolic fat stranding.
4.- Trace ascites in the left paracolic gutter and in the pelvis.
5.- No free air.

While in the ED he deteriorated rapidly, becoming obtunded along with worsening abdominal pain. VS were BP 77/29 mmHg, HR 112 bpm, RR 30 rpm. Given his neurological deterioration, the patient was intubated for airway protection. Central venous access was obtained. Meropenem was administered along with fluid resuscitation and vasopressors. Arterial blood gases obtained after intubation showed; pH 7.22, PaO_2_ 56.1 mmHg, PaCO_2_ 36.4 mmHg, HCO_3_ 14.6 mmol/L. The family was informed of the patient's critical condition. Consent was obtained for a diagnostic laparoscopy, possible exploratory laparotomy, possible ostomy.

**Fig. 3:**
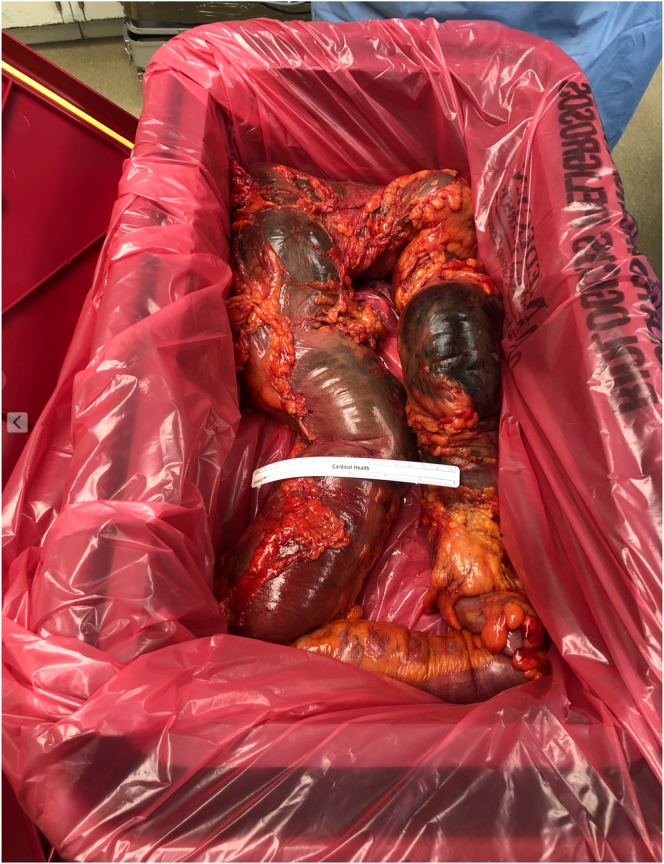
Extended left colectomy. Note the ischemia and necrosis as well as the colonic dilation.

In the OR an arterial line was placed. After time out the abdomen was accessed via Hassan technique through an infraumbilical incision. Obvious necrosis of the colon was visualized upon entry, leading to immediate conversion to an exploratory laparotomy. The entire left, and most of the transverse colon were dilated up to 12 cm, most of which was necrotic, especially at the sigmoid level. Proximal and distal transections were performed at the proximal transverse colon and sacral promontory respectively. Approximately 150 mL of free, murky fluid in the abdomen was noted. Dilation made the colon mobilization extremely difficult especially at the level of the splenic flexure. Intraoperative hypotension required an increase in vasopressor support and resolved after removal of the colonic segment. The right and proximal transverse colon looked viable. After ensuring hemodynamic stability an end colostomy was created, completing the operation. The segment of bowel removed can be seen in Fig. 3. Examination of the Hematoxylin and eosin stain on resected colonic samples can be seen in Fig. 5, Fig. 6, confirming focal colitis and necrosis.

**Fig. 4:**
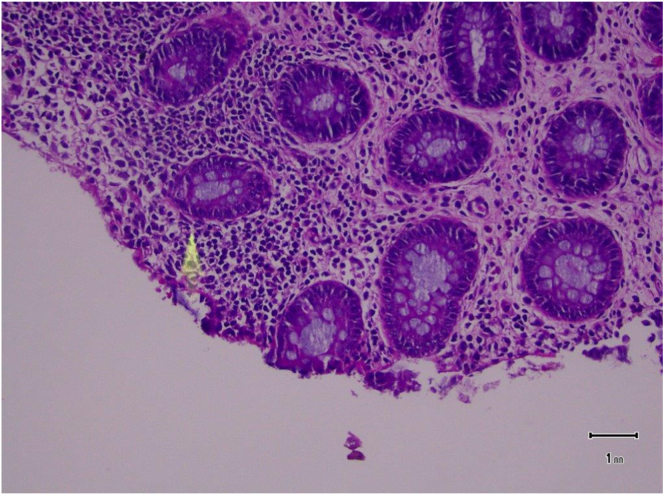
Benign mucosa with acute focal colitis without granulomas or crypt abscesses.

**Fig. 5:**
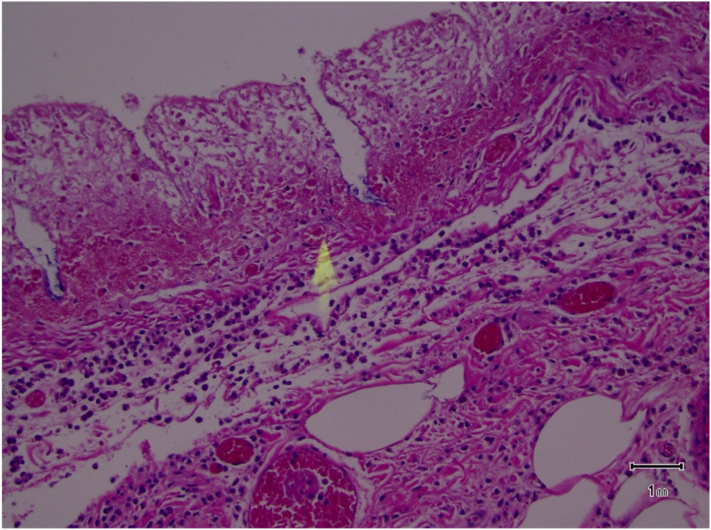
Benign mucosa with necrosis and suffused by red blood cells.

**Fig. 6:**
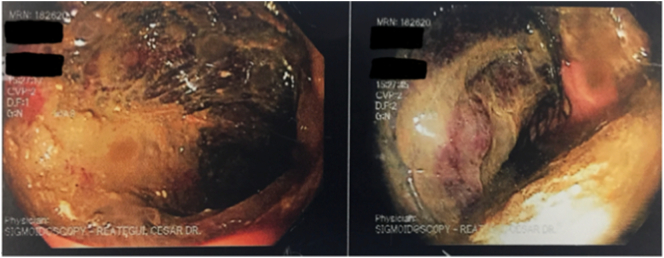
Flexible sigmoidoscopy in an elderly patient with Stercoral Colitis.

The patient was transferred to the ICU post-operatively. On post-operative day (POD) 3 vasopressor support was no longer required, with extubation occurring on POD 4. On POD 5 a clear liquid diet was started and he was transferred to the surgical floor. By POD 6, diet was advanced and colostomy began working. Discharge from the hospital occurred on POD 8.

He was seen in clinic on follow up 2 weeks after discharge. The patient reported doing well, tolerating regular diet, with colostomy functioning properly. Unfortunately, he was lost to follow up before planning colostomy reversal.

## 3 Clinical discussion

### 3.1 Presentation

Constipation can lead to fecal impaction causing SC. It most commonly affects elderly nursing home residents, chronic opioid users, and those with a degree of mental impairment. Physical examination can range from a non-distended, non-tender abdomen to peritonitis with full blown sepsis [4].

In the present case, the on-call surgical team recommended admission to internal medicine for colitis. However, rapid deterioration prompted an emergent surgical consultation for a second opinion. Surgery was performed by a board-certified general surgeon with fellowship in colorectal surgery. The patient's lack of history for vascular disease, or a clear embolic source left SC as the most likely etiology. This demonstrates the insidious nature in which NPSC can present and progressed rapidly to sepsis. Without prompt management, chances of a catastrophic outcome rapidly increase.

### 3.2 Imaging and pathology

Computer tomography (CT) is of paramount importance. Unal et al. described the CT finding associated with SC:

1.- Dilation of affected colon >6 cm.
2.- Wall thickening of affected colon segment >3mm.
3.- Pericolic fat stranding.
4.- Free air.
5.- Mucosal discontinuity.
6.- Free fluid.
7.- Pericolic abscess.

An affected length of >40 cm was associated with increased mortality [5]. In our case, the affected length involved the entire left and the distal transverse colon; far surpassing the >40 cm mark for increased mortality.

Wu CH, Huang et al. described 4 CT findings which exhibited direct correlation with mortality which included, in order of accuracy: dense mucosa (80.9 %) which results from mucosal hemorrhage, ascites (78.3 %), abnormal gas (78.3 %) which ranges from pneumatosis intestinalis to pneumoperitoneum, and perfusion defect (77.3 %) which can indicate a change from ischemia to infarction [6]. Diagnosis can be further confirmed with flexible sigmoidoscopy. Pressure on the rectal or colonic mucosa produces ischemia and necrosis that might lead to stercoral ulcer and perforation (Fig. 6).

In our case pathology revealed benign mucosa with acute focal colitis and necrotic changes (Fig. 4, Fig. 5), confirming the diagnosis.

### 3.3 Literature review

Using the word ‘Stercoral Colitis’, we performed a PubMed search for individual SC case reports and case series in the last 10 years. We found 3 case series and 22 individual case reports. The total amount of patients is 46, with 23 having colonic perforation. We compared the mortality rates in NPSC with sepsis to PSC.

Within the individual case reports were 13 cases of PSC with 3 fatalities [7], [8], [9], [10], [11], [12], [13], [14], [15], [16], [17], [18], [19]. There were 9 cases total of NPSC with 4 fatalities [4], [20], [21], [22], [23], [24], [25], [26], [27]. When the NPSC cases were stratified for sepsis, a total of 4 cases with 2 fatalities was yielded [24], [25], [26], [27].

Analyzing the case series, Cheng Wu et al. reported 5 mortalities in SC patients, from which 2 had PSC [28]. Evaluation of NPSC cases showed transmural necrosis in 2 of the 3 cases, and sepsis in all 3. All patients presented with acute abdomen and underwent surgery in this study. Unal et al. reported 6 patients with free air due to PSC, with 1 death [5]. Saksonov, et al. reported 13 patients with SC. Only 2 had PSC, both died. The 11 remaining cases were NPSC. Stratifying for sepsis yielded 4 cases, with 2 deaths [29].

When combining the cases of NPSC with sepsis from the case series published by Saksonov et al. and Cheng Wu et al., with individual case reports [24], [25], [26], [27] there was a total of 11 cases. Our case would make the 12th such case in the literature.

Combining the cases of PSC from the individual case reports [7], [8], [9], [10], [11], [12], [13], [14], [15], [16], [17], [18], [19], with the cases of PSC from the case series [5], [28], [29], there was a total of 23 cases. Table 2-1 illustrates the comparison in data obtained between PSC and NPSC with sepsis.

**Table 2-1:** Comparing mortality rates between NPSC w/sepsis to perforated SC.

	Deaths	Living	Total	Mortality rate
NPSC w/sepsis	7	4	11	63.6 %
PSC	8	15	23	34.9 %
Total	15	19	34	44.1

Sepsis alone carries a mortality rate ranging from 15 to 56 % [30], likely explaining the higher mortality rate in NPSC with sepsis (63.6 %) versus PSC (24.9 %).

### 3.4 Management

Patients without peritonitis can be managed non-operatively. This includes, at minimum, starting a bowel regimen, disimpacting fecal matter, and avoiding opioids.

Surgical management has been classically reserved for patients with PSC or failure of conservative management [31]. NPSC patients can rapidly develop colonic necrosis with sepsis; which is a surgical emergency. From our literature review, sepsis and lack of source control appear to be a greater determinant of mortality when compared to perforation status. The three most common locations for perforation are the anterior rectum proximal to the peritoneal reflection, the mesenteric border of the rectosigmoid junction and the apex of the sigmoid colon. Most perforate at the level of the antimesenteric border, perhaps due to diminished blood supply in this area. Surgical management consist of resection of the affected segment and colostomy with Hartmann's pouch. Colonoscopy role is not clear; it can be performed to ensure complete fecal disimpaction, assess the rectal and sigmoid colon mucosa and to disimpact fecalomas via fragmentation or with the help of loop wires.

The main weakness of this case report is the loss to follow up of the patient.

## 4 Conclusion

SC presents a clinical challenge. Early diagnosis and proper management are critical to avoid a fatal outcome. Patients presenting with NPSC w/sepsis carry a mortality rate >60 %.

## Funding

No sponsors.

## Ethical approval

No ethical approval needed.

## Consent

The patient was lost to follow up, consent could not be obtained. There is no identifying details in the manuscript.

## Author contribution

Study concept or design, data collection, data analysis or interpretation, writing the paper, by both authors.

## Registration of research studies

Not applicable.

## Guarantor

Cesar Reategui MD FACS.

## Declaration of competing interest

None.
